# The Importance of the Human Factor in Safety for the Transport of Dangerous Goods

**DOI:** 10.3390/ijerph18147525

**Published:** 2021-07-15

**Authors:** Sylwia Agata Bęczkowska, Iwona Grabarek

**Affiliations:** Faculty of Transport, Warsaw University of Technology, 75 Koszykowa Street, 00-662 Warsaw, Poland; iwona.grabarek@pw.edu.pl

**Keywords:** human factor, safety, human–systems interaction, hazardous materials, transport

## Abstract

This article discusses the issues related to the safety for the transport of dangerous goods by road. Research on accidents in transport unambiguously points to the human factor, which is the most responsible for causing accidents. Determining the causes of driver unreliability in the human−vehicle−environment system requires thorough research. Unfortunately, in this case, experimental research with human involvement is limited in scope. This leaves modeling and simulation of the behavior of the human factor, i.e., the driver transporting dangerous goods. The human being, because of its complexity, is a challenging element to parameterize. The literature presents various attempts to model human actions. Herein, the authors used heuristic methods, specifically fuzzy set techniques, to build a human factor model. In these models, human actions were specified using a verbal or linguistic description. The specificity of the fuzzy sets allowed for “naturally” limiting the “precision” in describing human behavior. The model was built based on the author’s questionnaire and expert research, based on which individual features were selected. Then, the traits were assigned appropriate states. The output parameter of the model was λ_L_—the intensity of human error. The obtained values of the intensity of the accident caused by the driver’s error were implemented into the author’s method of risk assessment. They constituted one of the factors determining the probability of an accident in the transport of dangerous goods, which allowed for determining the optimal route for the transport of these goods characterized by the lowest risk of an undesirable event on the route. The article presents the model’s assumptions, structure, and the features included in the model, all of which have the most significant influence on shaping the intensity of human error. The results of the simulation studies showed a diversified effect of the analyzed characteristics on the driver’s efficiency.

## 1. Introduction

The rapid development of transport has, on the one hand, increased the mobility of people and the transportation of goods, but, on the other hand, it has become one of the greatest threats of the 21st century [1,2,3]. That is why, in recent years, priority has been given to measures aimed at ensuring safety in both passenger and freight transport, and, in the latter case, in particular, the transport of dangerous goods. The package of measures include implementing relevant normative documents, obtaining certificates according to ISO standards, and setting up non-governmental institutions to ensure safety and control in transporting dangerous goods.

The Strategic Road Safety Action Plan of the European Commission for 2021–2030 aims to achieve zero road accident victims by 2050 (“Vision Zero”) [4]. There are many examples from all over the world of accidents or disasters involving dangerous goods in the literature [5,6,7,8,9,10]. In Poland, an average of 100 road accidents per year occur in the transport of these types of goods, with more than 1000 accidents in 2010–2019. Accidents involving tankers represent, on average, about 75% of all accidents per year. This trend remains stable and may even increase, as forecasts for the coming years show that truck traffic will increase [11]. Accident records kept by various state institutions, such as transportation technical supervision, fire brigade, or police, indicate the human factor as the main cause of errors leading to an accidents, in addition to other reasons, such as technical condition, infrastructure, and weather conditions. Apart from accidents or environmental disasters, there is often an uncontrolled release or unsealing of tanks, which, in extreme cases, may lead to a state of emergency or crisis in the area where it occurs. Statistics show that in Poland, 88–90% of dangerous goods are transported by road and only 10–12% by rail. Whereas in the EU, the transport of hazardous goods is as follows: inland transport (ADN) 6.8%, by rail (RID) 27.4%, and by road (ADR) 65.8% [11,12]. On the map of Poland, there are places characterized by a high intensity of accidents and incidents in road transport, including those involving goods with particularly hazardous properties. Particularly problematic places occur in four provinces: Małopolskie, Śląskie, Podlaskie, and Lubuskie. This is due to the location of chemical plants and the consequent increase in road traffic with dangerous goods. Dangerous goods in the 21st century can also be a weapon in the hands of terrorists. In July 2016, a terrorist of Tunisian origin drove a tanker truck into a group of people on a promenade in Nice, taking the lives of 87 people. Other such incidents or possible threat scenarios have been written about, including in [13,14].

From an analysis of the available literature and press reports, it appears that the carriage of these goods poses a significant hazard on the roads [15,16,17,18]. While the number of accidents is low compared with accidents involving passenger vehicles, the losses and danger are incomparably higher. Therefore, it is extremely important to have a comprehensive approach to analyzing the causes of dangerous events, taking into account the main elements of the transport process, namely: man (driver), technology (vehicle), and environment. As man is the weakest link in the system and at the same time the most important one, it is the one that should be given the most attention, followed by the other elements, assuming that each of them is more or less likely to cause accidents resulting in human losses.

The concept of human factors, and their importance to the safe performance of tasks, was recognized during World War II when it was recognized in the British Air Force that losses incurred in combat with the enemy were comparable to losses that resulted from a variety of human errors [19]. Chapanis first wrote that “pilot error” was the result of several components and could arise from poor control panel design. This was a challenge to contemporary thinking and highlighted the importance of design in reducing human error [20]. Three approaches currently dominate the human error literature. These are Norman’s (1981) error categorization; Reason’s (1990) classification of errors and violations; and Rasmussen’s (1986) classification of skill, rule, and knowledge errors. In road transport, Rasmussen’s theory is the closest, stating that the whole system and not just the operator performing the activity must be taken into account when analyzing an error that has occurred [21,22,23,24,25,26,27]. The systems approach maintains that most errors made in complex systems are caused by hidden or error-producing conditions (e.g., inadequate equipment and training, poor design, maintenance errors, and ill-defined procedures). This approach is particularly important in road transport.

Similarly, Wierwille et al. addressed the nature of error making systemically. He identified four basic groups of accident causation factors: human conditions and states (physical/physiological, mental/emotional, and experience/exposure), direct human causes (recognition errors, decision errors, and execution errors), environmental factors (highway-related and ambient conditions), and vehicle-related factors [28]. The driver-operator being part of the system may make errors: strategic errors, resulting mainly from a wrong decision made by the driver (e.g., concerning driving in difficult weather conditions, inoperative vehicle, or driving in an inappropriate physiological or mental state); tactical errors, resulting from inappropriate vehicle maneuvers while driving; and operational errors, resulting from lack of appropriate driving skills.

One of the theories for understanding human-driver behavior on the road in such a system is Ajzen’s theory of planned behavior (TPB) [29]. It explains driver decision-making as a result of intention and motivation. Therefore, some behaviors are unplanned and sometimes irrational, and result in accidents and related losses.

The driver in the system has a range of tasks to perform and is additionally loaded with a lot of information. A huge number of different stimuli reach the driver while driving. It is estimated that on a low-traffic urban road, it can be five stimuli per minute, while in dense urban traffic, even 120 stimuli in one minute [30].

In relation to the problem, the authors defined the system as a set of three elements: the driver transporting dangerous goods; the vehicle, a tanker for transporting dangerous goods; and the environment, i.e., the material working environment (D-V-E). The D-V-E system has specific tasks that are performed in the process of transporting dangerous goods. Due to the wide range of duties and the high level of responsibility for the transport of dangerous goods, the driver is required to have good knowledge of the regulations in the area of the goods transported, and to be conscientious and accurate in carrying out his/her work. However, despite mandatory training, the driver, as the weakest link in the system, makes mistakes due to the adaptation to work, the equipment of the workplace, and the state of the environment near and far.

**Figure 1:**
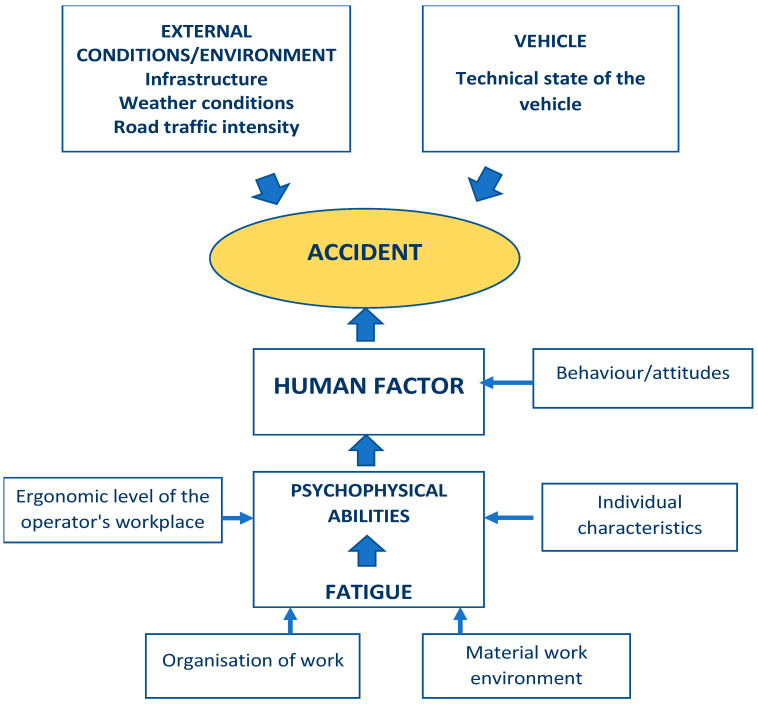
Factors potentially influencing probability of an accident.

Human error is therefore one of the main factors adversely affecting road traffic safety. As statistical data show, the defects of means of transport constitute a second most important group of issues influencing the abovementioned problem. The most popular vehicle used for the transportation of hazardous materials is a tank truck. Its common use results from the development of motorization and growing demand for fuels such as gasoline, diesel, or natural gas. Tank trucks receive road corridor permits from the transport technical supervision. It should be emphasized that the creation of a faultless DVE system is practically impossible, despite the supervision of technical devices and numerous compulsory trainings for drivers. Tank truck transportation of hazardous goods poses certain risk for man, and it is impossible to rule out the probability of an accident. Thus, the interactions within the DVE system should be analyzed and, based on the results, preventive measures should be adopted in order to reduce the risk within the transportation chain of hazardous materials. The above analysis made it possible to determine the structure of factors having a potential impact on the possibility of an accident This structure is presented in Figure 1.

As can be seen from the Figure 1, external factors such as infrastructure, i.e., the condition of the road, the surface, the weather conditions, or the amount of traffic, influence accidents. Most often, however, the human-operator is at fault. Incorrect driver behavior, which can potentially cause an accident, is due to a low ergonomic level of the driver’s workstation, which is influenced by the spatial structure, the seat, the legibility and placement of signaling or control devices, and the visibility of external signals. Moreover, fatigue, which is influenced by the driver’s age, psychophysical condition, stress, microclimate, vibrations, noise, number of working hours, or driving time, leads to a reduction in the driver’s psychophysical performance, which can cause an accident. Reduced performance is also due to the individual characteristics and predispositions of each person.

The study was the inspiration for the development of a new method of risk assessment taking into account the human factor and intended for use in the transport of dangerous goods. Consideration of the human factor as a key cause for an accident overcomes a shortcoming of the method, since, as we may read in the literature [31,32,33,34,35,36,37,38,39,40,41,42,43], no complex risk assessment method including the human factor has been elaborated so far. In this article, the main concept of the method described in the next paragraph is presented, with a focus on human factor modelling and its role in the transportation of hazardous materials. Heuristic techniques—particularly, fuzzy set methods—were used in the assessment of the human-driver.

## 2. Materials and Methods

In Poland there is no such a thing as a recommended risk assessment method that would take into consideration the specific nature of this kind of transportation. Statistical data that have been gathered for many years show that the majority of accidents taking place in Poland are caused by driver mistakes. This tendency is also present in other types of transport. The above described situation became an impulse for carrying out research on a new approach to the analyzed issues, taking into account numerous factors determining the level of risk in the process of the transportation of hazardous materials. These factors include the human factor (the most important one), technical factor (vehicle), environmental factor (workplace material environment), road factor, and a factor related to traffic intensity around fuel storage centers and refineries. Additionally, the method emphasizes the problem of human-driver fatigue, which limits efficiency and, consequently, decreases the level of road safety [44].

In this method, it was assumed that fatigue increases with time. The above premises were, therefore, the basis for undertaking research concerning a new approach to the problem in question, taking into account the many important factors influencing the level of risk in the process of transporting dangerous goods. Among them, one can distinguish the following: the most important one, i.e., the human factor; the technical factor, i.e., the vehicle; and the ambient factor, i.e., the material environment at the workplace, moreover, the road factor and the factor related to the intensity of vehicle traffic at fuel depots or refineries. In the proposed method, particular attention was also paid to the problem of human-driver fatigue, which affects the reduction of efficiency and, consequently, road safety [44]. An obvious assumption was made that fatigue increases with time. Actions aimed at minimizing the risk consisted of selecting the optimal route for the transport of dangerous goods, burdened with the least risk and thus the least losses. Due to the characteristics of the goods transported, losses may be of various types, such as human, ecological, and, as a result, financial.

In the analysis, risk was calculated as a product of the probability of an accident and the value of losses. An accident may lead to different scenarios of its consequences, and in the analysis they were interpreted as follows: an overturn of the vehicle with no additional consequences, an overturn of the vehicle with a leak, an overturn of the vehicle with a leak and a fire, and an overturn of the vehicle with a leak and an explosion.

The following hypotheses were formulated:

− it is possible to choose such a route that is characterized by the lowest risk of an accident during the transport of dangerous goods.
− the efficiency of the human factor changes depending on the level of individual characteristics (internal as well as external conditions (external characteristics).

The article focuses on proving the validity of the second hypothesis, which is related to the human factor.

A procedure was developed for the construction of a method that makes it possible not only to generate an optimal route for the transport of dangerous goods, taking into account the influence of individual factors, including human factors, on the probability of an accident, but also to estimate the risk. The method is strictly parameterized, which in turn allows it to be applied to the transport of all classes of dangerous goods. Although the article deals with modelling the influence of the human factor on the possibility of an accident, the structure of the whole risk assessment model is also described, taking into account other factors related to other elements of the operator−machine−environment system. The proposed accident risk model required the following steps:

1. To select factors of a different nature (human, technical, and environmental) affecting the probability of accident occurrence.
2. To take into account the influence of driver error on the probability of accident occurrence by means of a human factors model based on fuzzy set techniques.
3. Build a heuristic model to determine the severity of the accident due to human error. The model was developed based on factors considered “relevant”—generated from the analysis of the specificity of the system operation, procedural requirements of the type of transport in question, analysis of accident causes, and original surveys of drivers.
4. Division of the transport route into sections of different length (rectilinear or curvilinear).
5. To develop for each road section, defined according to the permissible speed limit, a road route environment.
6. Determine accident severity parameters due to technical condition of vehicles, other road users, human factors (e.g., driver fatigue), and traffic volume.
7. The value of intensity parameters λ were assigned to consecutive sections of a specified length, and the probability of occurrence of accidents caused by the above parameters was determined.
8. Definition of criteria enabling the categorization of human, ecological, and financial losses.
9. Assignment to each event scenario the level of human, ecological, and financial losses.
10. Determination for each categorized section of the sub-risk and the corresponding measure of loss value.
11. Construction of a simulation model enabling the selection of the route for the transport of dangerous goods from the start point to the endpoint in terms of minimization of human, environmental, and financial losses, whereby the route consists of different sections defined in points 5 and 6.
12. Carry out simulation studies.
13. Verification of the model on the selected actual transport routes of dangerous goods.

A simulator, based on the breath first search algorithm, was developed for the model, and a number of simulation experiments were carried out to select the optimal route depending on the objective function set, which is to minimize human, environmental, and financial take-offs.

The main assumptions for the risk model are presented below, while a detailed description is discussed in the work 

The following relation ties the probability of an accident and its consequences together:

(1) $$R=P_{a}\cdot L$$

where:

*R*—risk value;
*P_a_*—probability of accident occurring (in transportation of dangerous goods);
*L*—measure of losses.

Selection of the optimal transportation route for fuels from the starting point, e.g., the refinery to the final destination, i.e., a customer, can be done after the individual segments are characterized and their partial risk is defined. Thus, the formula for determining the risk value for a given segment will be (2):

(2) $$ER_{q}=P_{Xq}\cdot ES_{q}$$

where:

*ER_q_*—partial risk estimated for the *q*-th segment of the route;
*P_Xq_*—probability of accident in the *q*-th segment of the route;
*ES_q_*—expected value of loss on the *q*-th section of the route.

Whereas, the value of the total risk can be calculated as (3):

(3) $$ER_{T}=\overset{Q}{\underset{q-1}{\sum }}ER_{q}$$

where:

*ER_T_*—the estimated total risk for the transportation route;
*Q*—the set of segments constituting the route;
*ER_q_*—the fragmentary risk estimated for the *q*-th segment of the route.

This assessment of the accident probability in transport and the key factors influencing the probability are complex processes. As mentioned, the number of factors affecting the likelihood is significant, and they are often difficult to quantify. In the modeling process, a band model was proposed.

The probability of accident was defined for each *q*-the segment of the route, i.e., the connection between two adjacent vertices. It was assumed that the route is the sum of the analyzed segments. The first step in the band model was to assess the impact of the human, technical, and environmental factor on the probability of an accident in a particular *q*-th segment of the route. For this purpose, the parameter λ intensity was introduced. The concept of intensity, with reference to technical damage, is applied, i.a. in the reliability theory and is understood as the probability of object damage over a period of time (t, t + Δt), where t is high. Unlike the reliability theory, the events analyzed in this article are local in the sense that the time traveled or the road traveled by a vehicle during a simulation is far lower than the expected service life of the vehicle or the expected total distance traveled over the entire service life of a vehicle.

The notion of accident occurrence intensity and the probability density of an accident over “short” road segments, relative to the total distance covered by a car during its service life, have the same values and are, as such, in this case, identical.

The intensity is defined by the following relation:

(4) $$\lambda_{q}=\frac{P\left(x_{q}\right)}{x_{q}}$$

such that:

*λ_q_*—the intensity of accident occurrence on the *q*-th road segment;
*P*(*x_q_*)—the probability of accident occurrence on the *q*-th road segment;
*x_q_*—the *q*-th road segment [m].

**Figure 2:**
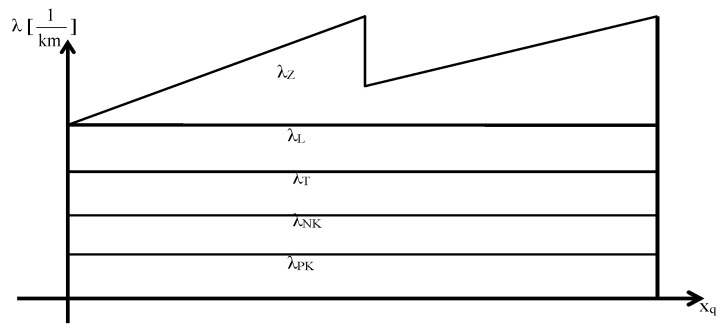
The band model of risk assessment.

The newly-introduced intensity parameter expresses the influence of the human, technological, and environmental factors on the probability of an accident occurrence. The influence of a human driver on the probability of accident occurrence is taken into account by the risk assessment model in two aspects. On one hand, the characteristics that could determine the driver’s efficiency and, if lowered, could cause mistakes, λ_L_. On the other hand, the influence of the driver’s fatigue on his performance are taken into account, adopting the linear relationship λ_Z_ in the time function at the first approach. On the Figure 2 shown the band model of risk assessment.

The following limitations have been imposed: λ_L_ is the intensity of accident occurrence caused by the driver’s mistake, 0 ≤ λ_L_< 1,

λ_Z_—the intensity of accident occurrence caused by the driver’s fatigue_,_ 0 ≤ λ_Z_< 1;
λ_T_—the intensity of accident occurrence caused by a technological factor, 0 ≤ λ_T_< 1;
λ_NK_—the intensity of accident occurrence caused by conditions over which the driver has no influence, i.e., caused by other users of the roads, 0 ≤ λ_NK_< 1;
λ_PK_—the intensity of accident occurrence near fuel storage facilities, 0 ≤ λ_PK_< 1.

In this model, only λ_Z_ changes over time. Increasing the duration of the route along with the increasing the total distance covered has a significant impact on the efficiency of the driver, which decreases as his fatigue increases. In the majority of cases, the values of the intensity for a specific “segment” are assumed to be constant. The values may depend on, for example, the type of road segment (road conditions, which is dependent on e.g., the maximum speed limit) or the traffic congestion level. Parameter λ_PK_, assumes a constant value when the vehicle is in the proximity of a fuel storage facility, or a value of 0 if otherwise.

This value was determined based on the data collected by the central statistical office, transport technical supervision service, state fire service, and police headquarters.

The values of the intensity of accident occurrence for specific factors assigned to their own “segment” *x_q_* constituted the base of calculations of the probability of accident occurrence in the transportation of hazardous products for the given *x_q_* “segment”, (5):

(5) $$P_{wq}=\{\begin{array}{c} \overset{x_{mn}}{\underset{0}{\int }}\left(\lambda_{L}+\lambda_{T}+\lambda_{N}+\lambda_{PK}+\lambda_{Z}\left(x\right)\right)dx\\ 1,\;if\;\overset{x_{mn}}{\underset{0}{\int }}\left(\lambda_{L}+\lambda_{T}+\lambda_{N}+\lambda_{PK}+\lambda_{Z}\left(x\right)\right)dx>1 \end{array}$$

such that:

*Px_q_*—the probability of an accident occurring on the *q*-th route “segment”;
*x_q_*⇔*x_mn_*—the length of a segment between the points m and n.

For the purposes of this article, the modeling of the λ_L_ parameter, or the intensity of an accident as a result of a driver’s mistake, is described in more detail. This factor, in terms of the methods of risk assessment in the carriage of dangerous goods, is often overlooked. On the other hand, there are a number of reliability methods discussed in Section 2, however they cannot be applied due to the lack of adequate datasets on the probabilities of driver mistakes. Therefore, the existing methodological gap in this area was an inspiration for the research on modeling of the impact of human factor on accident probability and, ultimately, its inclusion in the risk assessment model for the road transport of dangerous goods.

### 2.1. Human Factor in the Transport

The statistically confirmed significant contribution of man to the causes of accidents requires attention to be paid to the specifics of this profession. The profession of a driver-operator falls into the category of difficult and dangerous (ADR 2019–2021) [45]. In the road transport system, the human factor plays a decisive role and has two functions: performs as a co-author of road traffic and as a road user. The driver carrying hazardous materials is exposed to numerous factors, which deteriorate their ability of job performance and even lead to the deterioration of their health. A driver’s safe functioning within road traffic systems depends greatly on their psychophysical characteristics, social adaptability, driving etiquette, and ability to deal with complex situation, such as driving a vehicle. Carrying out a test verifying drivers’ vocational usability (the so called “professional selection”) is a duty of specialized services [46,47]. The methods of such a selection are thoroughly developed. The final decision of whether to offer a job is made based on the required documents and appropriate professional courses certificates, medical examinations, and observation during a trial period. According to the literature, selecting only those drivers with certain predispositions is a highly effective method of reducing the risk of accidents in the transport of hazardous materials.

Professional profile of the driver carrying hazardous goods includes the following [46,47]:

(1) Sensorimotor efficiency:
  (a) Critical features: visual acuity, color sensitivity, stereoscopic vision, night vision, sense of smell, visual−motor coordination, quick reflexes, perceptiveness, and dexterity;
  (b) Useful features: good hearing, sense of balance, and sense of touch.
(2) Abilities:
  (a) Critical features: powers of concentration, ability to multitask, imagination and creative thinking, and technical skills;
  (b) Useful features: good memory and logical reasoning.
(3) Personality
  (a) Critical features: long-term endurance, self-control, ability to work in isolation, ability to work under monotonous conditions, courage, and precision;
  (b) Useful features: emotional endurance, ability to follow instructions, readiness to work under difficult environmental conditions, perseverance, and patience.

The analysis of the transport systems shows that the technical machine operator makes mistakes, despite his abilities that meet their professional profile. Those mistakes are due to not only individual features, but also to workplace conditions, such as existing vibrations, noise, and microclimate. Monotony constitutes a significant element of a truck driver’s work process. The higher the level of monotony, the lower the driver’s alertness, which may pose a risk to the life and safety of the driver and other road users [48]. Driving at night, which entails straining a driver’s eyes, decreases vigilance. The modern constructions of a driver’s cab can improve an operator’s work conditions: comfort at work increases and physical effort related to driving is reduced. However, these improvements do not manage to isolate the truck driver completely from vibrations and noise created by the vehicle’s movement. The impact of the factors mentioned grows with the increase of speed and the number of road users. As a consequence, a driver’s fatigue rises quickly. Weariness leads to a decrease of physical efficiency, leg−hand coordination disorder, and limitation of the visual field. As an effect, sleepiness and apathy increase. Driver fatigue also has an influence on reaction stability, speed and scope of perception, attention, and reaction time. Taking into consideration the complexity of the situation in which the driver carrying hazardous materials operates, we can distinguish various types of fatigue, for example: muscle fatigue (related to driving posture and holding steering wheel), sensory fatigue (reduced capacity to answer to stimulation), and mental fatigue (decrease of cognitive performance caused by constant focus on a task and monotony of driving conditions). The consequences of driving despite appearing fatigued may be dangerous not only for drivers themselves, but also for other road users. The highest share of accidents caused by drivers’ fatigue was recorded on highways A2, A1, and A4, as well as on national roads n°2, 3, 17, 18, and 22. The conclusion from the above-mentioned arguments supports a thesis that it is practically impossible to protect drivers carrying hazardous materials from temporal information overload, emotional tension, or real and unexpected situations. It should be noted that to a significant degree, the driver is responsible for the safety of the D-V-E system, although they are just a component of the greater whole. Therefore, a penetrating analysis of this component and the actions aimed at strengthening it will reinforce the whole system. The development of the human factor model required, as envisaged, the selection of characteristics that influence the intensity of the occurrence of an accident due to human error.

An analysis of the driver’s performance has allowed for determining the structure of factors with a potential influence on the probability of an accident. In the years 2012–2018, the factors mentioned were evaluated through a survey by drivers carrying hazardous materials. The evaluation was carried out among 250 drivers in various age groups, with different work experience, and who drove different types of vehicles used for the transport of hazardous goods. The respondents between 36–49 years old constituted the most largest age group. As an average, these drivers were driving between 10,000–12,000 km per month. Their vehicles were relatively new—they were produced in the years 2009–2017. The anonymity of the survey gave the respondents the opportunity to openly discuss the existing difficulties and risks related to their workplace. The data gathered through evaluation were statistically analyzed with the use of R Development Core Team Software R 3.6.3 (Holding the Windsock). The results show that the factors considered by drivers as being arduous included the following: vibrations (95%) and noise (73%), that is, vibroacoustic environment conditions; night shifts (52%); time pressure (59%); monotony (50%); and stress (56%). Based on the research results and specialist literature, the most significant factors from the point of view of ergonomics and safety in the transport of hazardous materials were selected. Subsequently, a fuzzy model was created, which allowed for generating the intensity of accidents caused by driver mistakes (λ_L_), described in detail in the following paragraph.

### 2.2. Assumptions of Heuristic Model

The aim of the modelling was to determine the influence of selected factors, referred to as features in this model, on the intensity of accidents caused by driver mistakes. The features were selected based on expert-based surveys and literature. The Mamdani model was used for model creation [49].

The procedure of creating the model included the following:

1. Selection of features, that is, external characteristics formed by organizational and technical conditions, such as, working time, skills, vibrations, noise, knowledge of procedures, and internal psychological and physiological characteristics, such as stress, age, time of day, and monotony.
2. Assigning certain levels of selected features with their corresponding fuzzy sets, that is, monotony (low, mid, or high), knowledge of procedures (good or poor), skills (good or bad), working time (standard or overtime), vibroacoustic conditions (bothersome or non-bothersome), stress (low, mid, or high; treated as a “submodel” of internal characteristics), age (young, middle, or mature), and time of day (day or night).
3. Assigning trends in the formation of λ_L_ (the intensity of accident occurrence due to human error) in the form of ideograms as keywords:
  Very small ↓ ↓
  Small ↓
  Medium ------
  Large ↑ ↑
  Very large ↑ ↑

**Figure 3:**
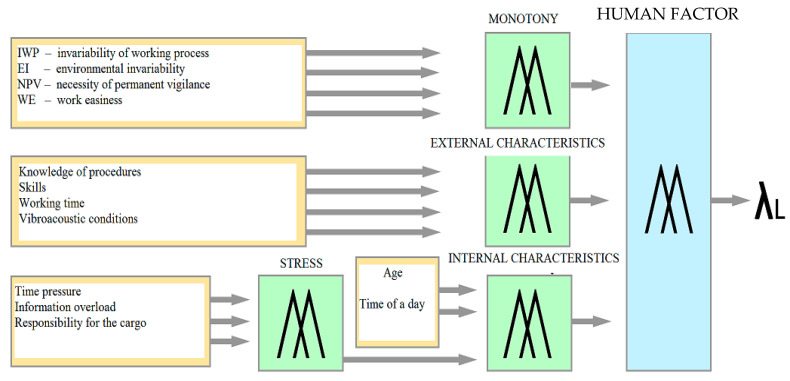
Heuristic model of intensity of accidents caused by driver mistakes.

The analyzed structure is presented in Figure 3.

The input parameters of the human error accident intensity model are monotony, external characteristics, and internal characteristics. According to the formulated assumptions, monotony was described by an invariability of the working process, environmental invariability, necessity of permanent vigilance and focus, and work easiness. External characteristics were described by working time, knowledge of procedures, vibroacoustic conditions, skills. Internal characteristics were described by stress, age, and driving time, with stress depending on time pressure, information overload, and responsibility for the cargo. The trait of stress is a fuzzy submodel of the internal trait model. The output of the model is the intensity of accident occurrence due to human error.

All of these input parameters can affect a driver performance in different ways depending on their level and configuration. They can become distractors or, on the contrary, positively influence driver fitness, which, in this case, was defined as the intensity of accident occurrence due to human error. The influence of these characteristics on fitness was described using a linguistic model, which is usually used to describe those phenomena and concepts that are ambiguous and imprecise in nature.

In heuristic modeling, numerical values that are “measures” of a given feature should be assigned to particular components. Due to the numerical effectiveness of the algorithms described in the following section, the value of the quantity λ_L_ occurring in the model, i.e., the intensity of an accident that occurred as a result of human error, was normalized according to the formula:

(6) $$\lambda_{L}\;=\;\frac{x-x_{\min}}{x_{\max}\;-\;x_{\min}}$$

where:

*λ_L_*—intensity of accidents caused by a driver’s mistake;
*x*—current value of a real variable;
*x*_min_—minimum value of a real variable;
*x*_max_—maximum value of a real variable.

As above mentioned, the standardization of variables is appropriate from the point of the view of numerical calculations (scope of change for each variable is the same and, as a consequence, the problem is numerically very well defined). The Equation (1) implies that reverse operation (change from standardized to real variables) is explicitly determined. The minimum and maximum values of a real variable were calculated based on the data from the state fire service, central statistical office, and own research. The analyzed data referred to the local chemical and ecological threats involving tank trucks from the last 5 years, the number of tank trucks registered in the same period of time, and the average number of annual working hours of tank trucks’ drivers. In order to determine λ_Lmin_ and λ_Lmax_ of the above mentioned period of time, the years with the smallest and the largest numbers of dangerous incidents were chosen and combined with the number of registered tank trucks in a given year. The λ_L_ parameter takes into account only the incidents related to the probability of an accident caused by a driver’s mistake. Due to the lack of detailed databases, the analyses were based on the level of men’s participation, expressed in %, as a main cause of chemical and ecological accidents according to the National Headquarters of the State Fire Service. Based on the analysis of the available data, the intensity of accidents caused by human mistakes λ_L_ was determined as the following interval: (0.0000016; 0.0000021). In the case of qualitative variables, this method requires assigning a value that ranges in degree from 0 to 1 to a given feature. This value refers to “expert” evaluation of the feature’s intensity. Values assigned to particular features, that is, quantization levels, are presented on the Table 1.

The selected features were assigned by fuzzy sets and their corresponding membership function (MF) [50]. First, the monotony model was defined by four factors determining its level, then the model of external factors and the stress submodel, included in the model of internal factors, were determined. The features, together with their levels, formed grounds for a linguistic-based heuristic model, formulated as an implication. A fragment of the rules referred to the stress submodel has been presented below:

“If time pressure is low and the level of information overload is low and responsibility for a cargo is small then the stress level is low.

If time pressure is low and the level of information overload is low and responsibility for a cargo is big then the stress level is low.

If time pressure is low and the level of information overload is high and responsibility for a cargo is small then the stress level is low.

If time pressure is low and the level of information overload is high and responsibility for a cargo is big then the stress level is medium...”

The creation of remaining models was analogous, and was referred to as monotony, external, and internal characteristics. Their linguistic form was applied to the model of intensity of accidents caused by driver mistakes.

### 2.3. The Structure of Fuzzy Model and Its Numerical Implementation

**Figure 4:**
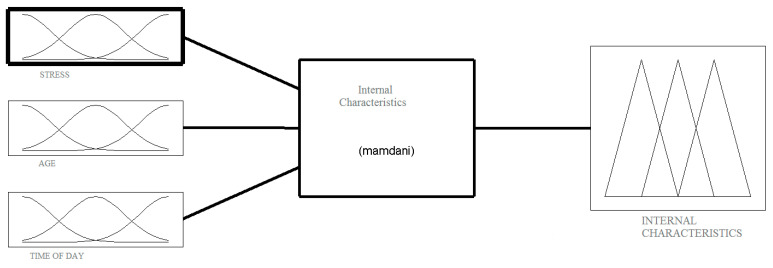
Mamdani model for internal characteristics simulation.

The heuristic model, described in the previous paragraph, was numerically implemented in a MATLAB/Simulink environment. The model includes three structures, in accordance with Figure 2. Fuzzification is the main process in fuzzy modelling in which the crisp quantities are converted to fuzzy ones. The conversion into fuzzy values is represented by a membership function. There are various methods of assigning membership values or the membership functions to fuzzy variables. Membership value ranges in degree between 0 and 1, and it represents the degree of membership of each input linguistic variable to a corresponding fuzzy set. In the Mamdani model, the Gaussian function was adopted as a membership function [51]. Other available membership functions, that is, triangular and trapezoidal, had no significant influence on the results. The Mamdani model for internal characteristics simulation are shown as an example in Figure 4.

**Figure 5:**
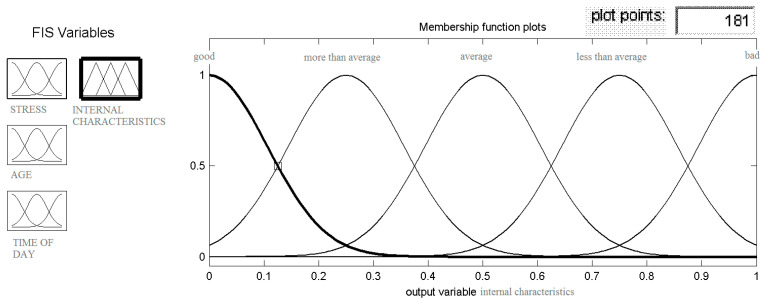
Membership function for internal characteristics.

The total of membership functions determine the number of quantization levels adopted for the input signal. For each feature the Gaussian membership function was applied, as shown in Figure 5.

The internal characteristics were developed at five levels, in accordance with the assumptions. Analogous operations were performed for each of model’s structure. As can be seen, the model has various elements that can be modified and it is precisely this flexibility that allows for reflecting the reality. Nevertheless, it entails laborious and expensive experimental research, as a drivers themselves constitute the main object.

## 3. Results

**Figure 6:**
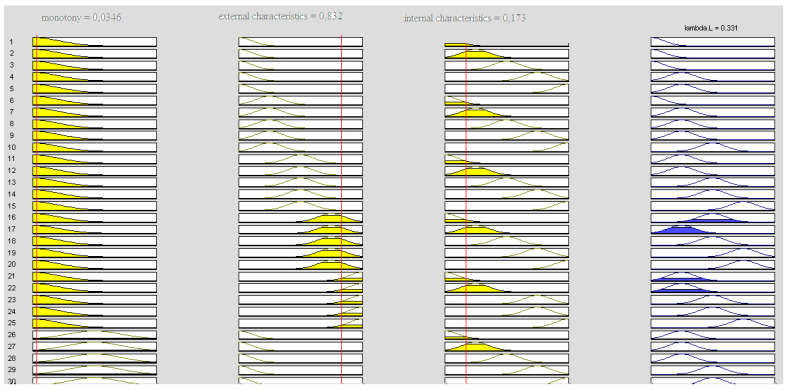
Visualization of implication rules for simulation λ_L_.

This section gives example results of the simulation of the effect of individual characteristics on the intensity of human error accident occurrence using the Mamdani model. Figure 6 shows an example visualization of 29 of the 75 implication rules of the intensity of human error accident occurrence, with assumed settings for monotony, external factors, and internal factors.

For this simulation, the intensity of the accident due to human error was 0.331 (in standardized variables

**Figure 7:**
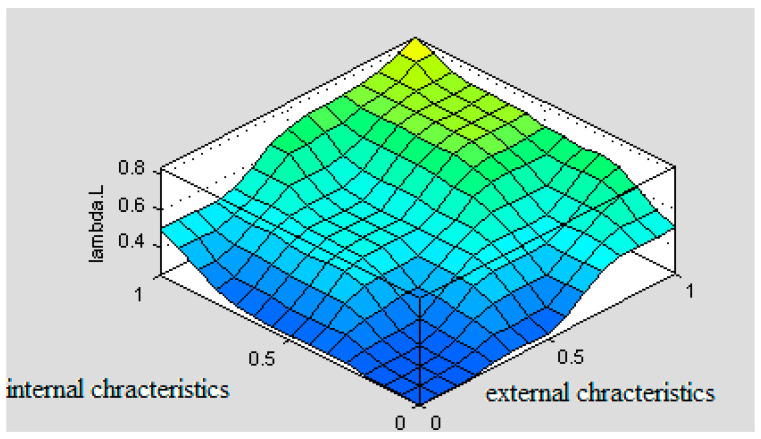
Simulation results for intensity of accidents caused by human mistakes depending on external and internal characteristics.

Taking different values of the input parameters (Figure 6) will result in a different value of the output parameter lambda L. Figure 7 shows an example of the effect of both groups of features (external and internal) on the human factor, assuming that monotony is essentially absent. The shape of the plot is derived from the developed linguistic model, with values given in standardized variables, taking values from 0 to 1. Depending on which values the internal and external features take, lambda L will vary.

Under the above assumption, the course of lambda L changes for internal features and takes a shape indicating that they influence the intensity level of λ_L_ to a greater extent than the external factors. Assuming, that the standardized value of both parameters is the same, e.g., 0.6, it follows from the graph that the group of internal features causes a greater increase in lambda L than the group of external features.

The simulation model, which is a numerical implementation of the linguistic model in the Matlab_Simulink ver. 7.5 environment, makes it possible to analyze the influence of the input parameters, taking different values (from 0 to 1 in standardized variables) on the intensity of accident occurrence as a result of driver error. At the same time, it is possible to analyze changes in λ_L_ by changing the configuration values of the input parameters. Because the object of research is a human being, it is impossible to study such different situations in real conditions. Computer modelling and simulations are an effective research tools for determining the relationship in the driver−vehicle−environment system. The estimation of the parameter λ_L_ is a component of a model called the band model, by means of which it is possible to generate the probability of an accident occurring in the road transport of dangerous goods, and consequently to propose the optimal route for the transport of dangerous goods with the lowest risk of an adverse event.

## 4. Conclusions

Dangerous goods constitute an essential segment of the transport market and are necessary for the functioning of industry and urban agglomerations. The transport of hazardous goods, regardless of the technical means used, poses a potential safety risk. Measures to reduce this risk are therefore a priority. However, it should be added that even the most stringent requirements can be of little effect without a risk assessment that takes account primarily of the human factor, i.e., the main offender of road accidents. It is impossible to carry out tests under natural conditions, primarily because of their limited scope on the human body. In addition, there are constraints relating to financial and organizational capacities. On the other hand, it is the driver who is the most unreliable element in the whole system, and therefore, determining their capabilities and the most favorable working conditions for their efficiency is one of the most important research problems.

Simulation studies have shown the truth of the second hypothesis, i.e., that the efficiency of the human factor varies depending on the level of individual characteristics (internal), as well as external conditions (external characteristics). Appropriate shaping of working conditions, work organization, and professional selection can reduce the intensity of driver error.

The literature contains research results [52,53,54,55] on the causes of accidents, namely driver performance resulting from a single impact of the factors studied or several simultaneously. There are no studies on driver’s efficiency that consider a set of elements reflecting the nature of a driver transporting dangerous goods to the extent required by the model developed by the authors. No studies clearly define the relationship between the effects of single factors and their collective impact. The authors attempt to model the intensity of an accident caused by driver error and, thus, the selection of the optimal route allows for a more in-depth analysis of the issue and constitutes a step closer to modeling the relations occurring in the whole system.

Full verification of the developed model can be achieved through:

1. an “active” experiment;
2. a “passive” experiment.

The “active” experiment requires access to a truck simulator equipped with an appropriate measuring system. It should then be possible to regulate the tested parameters of the human factor, the constructional-technical and material working environment, and count the errors committed. Due to the participation of humans in the experiment, working conditions should be shaped with great care. A “passive” experiment is conducted under regular vehicle operating conditions. It requires the vehicle to be equipped with apparatus for monitoring the running process. The monitoring concerns both the factors under investigation and the driver’s behavior in selected situations.

Carrying out experimental (complete) verification depends on the financial outlay and organizational capacity of both the university and the transport companies. The results obtained from the simulations make it possible to determine many relationships between individual characteristics.

Another issue is method validation. By definition, modelling and simulation validation is the process of determining the extent to which the model, simulation, and associated data are accurate representations of the real world for the intended applications. It requires accurate real-world data and testing of the method with potential users, which, for formal reasons, has not yet been done.

Results obtained from simulation studies of the developed model identified many relationships between individual characteristics and their impact on driver performance. It was shown that depending on the state of the input parameters, the efficiency changes, which is a characteristic of the human factor. The linguistic variables, both input and output, have been expressed in standardized values, which allows, as reliable real parameter values are obtained, for modifying the model from this point of view. As mentioned earlier, data on accidents and their causes are collected by different institutions and usually according to different criteria. The human factor model was implemented into the author’s risk assessment method and constituted one of the factors for determining the probability of an accident in the transport of dangerous goods, which makes it possible to determine the optimum route for the transport of these goods characterized by the lowest risk of an undesirable event occurring along the route. A number of routes connecting selected cities in Poland were evaluated, and the best one was chosen from the point of view of minimizing the risk of an accident. Carrying out this type of research and computer experiments gives great cognitive opportunities and can be an effective tool to study the links in the K-P-O system, which are difficult to measure in real conditions. The study of the human factor as the basic and most unreliable element in the transport system requires the search for and application of various methods that will most accurately reflect its functioning in different working conditions. Undoubtedly, the application of AI methods will bring us closer to solving problems related to such a complex matter as the human being. As long as there will be no high autonomous transportation systems, the problem of human-operator unreliability will always be present.

## Figures and Tables

**Table 1:** Extent of change in the values of the analyzed characteristics.

Factor /Input/	The Values of Linguistic/Fuzzy Sets Value in Range <0; 1>				
	0	0.25	0.5	0.75	1
IWP—invariability of working process EI—environmental invariability NPV—necessity of permanent vigilance WE—work easiness	small				big
Monotony	low		mid		high
Knowledge of procedures Skills Working time Vibroacoustic conditions					
	good				poor
	good				bad
	standard				overtime
	non bothersome				bothersome
External characteristics					
	good	more than medium;	Medium;	less than medium	bad
Time pressure Information overload Responsibility for cargo	low				high
	low				high
	small				big
Stress	low		mid		high
Age Time of day	young		middle		mature
	day				night
Internal characteristics	good	more than medium;	Medium;	less than medium	bad

## Data Availability

Not applicable.
